# Diagnostic Delay of Spinal Tuberculosis Causing Medullary Compression

**DOI:** 10.1155/2024/5118600

**Published:** 2024-05-03

**Authors:** Krestine Corydon, Matilde Bjørn Ørum, Kristoffer Backman Nøhr, Kristina Öbrink-Hansen

**Affiliations:** ^1^Department of Internal Medicine, Gødstrup Regional Hospital, Herning, Denmark; ^2^Department of Infectious Diseases, Aalborg University Hospital and Department of Infectious Diseases, Aarhus University Hospital, Aarhus, Denmark; ^3^Department of Radiology, Aarhus University Hospital, Aarhus, Denmark

## Abstract

We present a case of a Philippine woman in her late twenties, diagnosed with spinal tuberculosis after surgical intervention due to medullary compression. The diagnosis was preceded by four months of unexplained back pain. Differential diagnoses included ulcer, liver-gallbladder disease, musculoskeletal causes, and cancer. This case highlights the importance of considering tuberculosis as a differential diagnosis in patients from high-endemic areas to avoid diagnostic delay and the risk of disease progression.

## 1. Introduction

Tuberculosis (TB) is caused by the bacteria *Mycobacterium tuberculosis (M. tuberculosis)*. It is common worldwide with 10.6 million cases reported in 2021, but the incidence varies between countries. High-endemic areas include India, Indonesia, China, the Philippines, Nigeria, Bangladesh, and DR Congo [1]. Pulmonary TB is the most common presentation, but *M. tuberculosis* can affect virtually any tissue in the body causing extrapulmonary tuberculosis (EPTB). In Denmark, the most common site of EPTB is the lymph nodes and the pleura followed by spinal TB [2].

Spinal TB primarily affects the thoracic and thoraco-lumbar region. The most common symptoms are back pain, weight loss, fever, night sweats, and neurological deficits [3, 4]. The diagnosis of spinal TB is often delayed. This is due to a number of reasons, including subacute presentation, multiple differential diagnoses, and in less-resourced settings a lack of diagnostic possibilities [5]. Gold standard method for spinal TB diagnosis is microbiological identification *of M. tuberculosis* via Computed Tomography- (CT-) guided biopsy, which might not be available in less-resourced settings. In both resource-rich and less-resourced settings, doctor's delay may contribute to diagnostic delay [6]. The treatment of spinal TB consists of long-term anti-tuberculous medication, and in some cases also surgery. Despite treatment, nearly half of the patients with spinal TB suffer from sequelae; primarily back pain, but neurological complications ranging from impaired movement to tetraplegia are also seen. Furthermore, kyphosis has been reported as more common sequelae six months following treatment [3, 7].

We describe a case of spinal TB with medullary compression that illustrates diagnostic delay in a resource-rich setting.

## 2. Case Presentation

An immune-competent, Philippine woman in her late twenties was seen in the emergency department with strong pain under both rib curvatures with a maximum on the right side. Two days before, a gastroscopy revealed small ulcerations and a positive *Helicobacter pylori* quick-test, and eradication treatment with relevant antibiotics in combination with a proton pump inhibitor was initiated. This was all preceded by four months of progressive, intermittent pain from the right rib curve, radiating towards the back and shoulder blades. The patient's general practitioner interpreted the pain as of musculoskeletal origin and referred her to physiotherapeutic treatment. Other differential diagnoses considered included diseases in the liver and biliary system, and the patient was referred to an abdominal surgeon for further investigation.

**Figure 1:**
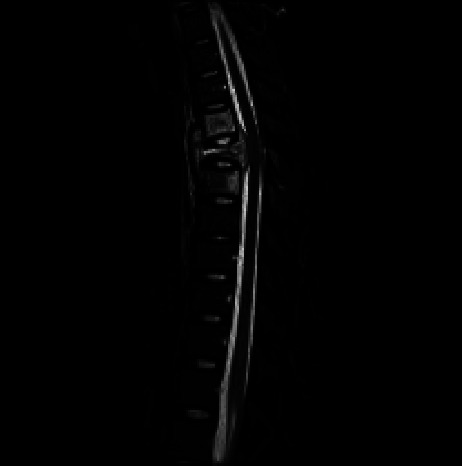
Sagittal short tau inversion recovery (STIR) sequence demonstrating destruction of vertebral body T6 accompanied with oedema. Abscess formation extends into the paravertebral soft tissue as well as the epidural space in the anterior part of the spinal canal. Intramedullary T2 hyperintensity suggests compressive myelopathy. Due to the destruction of both the anterior and middle vertebral columns, the segment must be considered unstable. Adjacent level T2 hyperintensity suggests infectious spread without endplate destruction.

At the time of admission, laboratory results showed low grade inflammation with C-reactive protein (CRP) of 20 mg/L, sedimentation reaction (SR) of 62 mm, and leucocytes within normal range. Alanine aminotransferase (ALAT) was 50 U/L, and the haemoglobin level was 7.7 mmol/L. The patient tested negative for human immunodeficiency virus (HIV). Because of strong pain in the chest region with radiation towards the shoulder blades, a CT scan was performed to rule out aortic dissection. No dissection was found; instead, the CT scan depicted an unstable fracture of T6 with surrounding soft tissue mass and suspected spinal cord compression. The patient was however neurologically intact. Subsequently, a Magnetic Resonance Imaging (MRI) scan of the spine and pelvis showed severe destruction of vertebral body T6 with an almost entire loss of height both anteriorly and posteriorly and a surrounding soft tissue mass (Figure 1). The ventral epidural soft tissue component was seen displacing the thecal sac, and intramedullary T2 hyperintensity was suggestive of compressive myelopathy. There was oedema in adjacent vertebral bodies without endplate destruction. The scan also showed oedema in C2 and T1 without bone destruction and multiple similar lesions in the pelvis.

The patient underwent surgical intervention with decompression, laminectomy, and spinal fusion with pedicular screws inserted in T3, T4, T5, T7, and T8. During the procedure, a pocket with pus-like material was emptied. The material was sent for microscopy, bacterial cultivation, 16sPCR, specific PCR for *Staphylococcal aureus*, and to the Danish national reference laboratory for TB diagnostics (microscopy, PCR, and cultivation). In the following days, the patient was investigated for malignant metastatic disease. A CT scan of the thorax, abdomen, and pelvis showed a solid process (sized 2.2 × 2 cm) at the left ovarium, and a subsequent gynaecological examination with ultrasound and smear ruled out malignancy. A positron emission tomography- (PET-) CT scan showed highly increased fluorodeoxyglucose (FDG) uptake in several areas in the vertebrae and pelvis as well as highly increased FDG uptake in enlarged lymph nodes in the mediastinum and in retroperitoneum. At this time, elaborate history revealed five kg weight loss, night sweats, and a sensation of fever during the past four months. The patient had immigrated from the Philippines to Denmark six years earlier. A second degree relative had been treated for TB when the patient was a child.

A bone biopsy obtained during the surgery showed inflammation with necrotizing and non-necrotizing granulomas. Interferon-gamma Release Assay was positive. However, *M*. *tuberculosis* polymerase chain reaction (PCR) was negative and there were no acid-fast bacteria found by microscopy of the material. Two weeks after surgery, *M. tuberculosis* was confirmed by cultivation. No resistance mutations were found.

The patient initiated anti-tuberculous treatment according to standard protocol with ethambutol, isoniazid, pyrazinamide, and rifampicin for two months, followed by rifampicin and isoniazid for another seven months. The treatment was supplemented with pyridoxine. During treatment, the inflammatory markers and ALAT were normalised. A PET-CT scan five months after the treatment was initiated which demonstrated complete regression of FDG uptake in the bones and lymph nodes and also morphological signs of healing. By the end of treatment, the patient continued to have discomfort with impeded sensory and also neurogenic pain beneath the right breast, but with no physical limitations.

## 3. Discussion

TB remains a leading infectious cause of death worldwide and is still a disease of significant burden with a total of 10.6 million new cases in 2021. Incidence varies according to geography, and in 2021 eight countries accounted for more than two-thirds of all cases, here among the Philippines [8]. In Denmark, the incidence is low and has been decreasing the last 20 years with 3.9 cases per 100.000 inhabitants in 2022 [9]. The majority of cases were diagnosed in patients of non-Danish origin. Pulmonary TB is by far the most common presentation for both patients of Danish and non-Danish origin. However, EPTB occurs somewhat more often in patients of non-Danish origin, which is in line with a previous Danish study by Holden et al. [2]. Spinal TB is a rare condition in developed countries. In Denmark, 153 cases were reported from 1994 to 2011 and 83.3% of these cases were immigrants [3]. Back pain is the most common symptom [10] but is also a very common symptom in the population in general, as 7.5% of all contacts to the general practitioner involve back pain [11].

In our case, the patient's primary complaint was back pain. She was immune-competent and inflammation markers and liver parameters were only modestly elevated, which probably contributed to the fact that four months passed from the onset of symptoms until the time of diagnosis. During these months, diagnostic considerations included musculoskeletal pain, disease in the biliary system, ulcer, and malignancy. In Denmark, the median duration from contact to the hospital until the diagnosis of spinal TB is 19.5 days, and the median duration of symptoms is 120 days [3]. This is in line with a systematic review of case series, which reported the mean average time until the diagnosis to be 6.5 months [10].

In a Dutch study regarding spinal TB, with resource settings similar to Denmark, Ijdema et al. reported a median doctor's delay of three months, which is in line with our case [12]. Interestingly, Ijdema et al. did not observe any association between occurrence of risk factors for TB and doctor's delay and even found an increased doctor's delay in patients with the classical triad of symptoms typical for spinal TB (night sweats, back pain, and weight loss). In contrast, Holden et al. found that Danes had a significantly longer doctor's delay compared to immigrants but also that immigrants had significant longer patient delay and therefore might be in a more advanced stage at time of presentation to the healthcare system [2]. Nonetheless, it underlines that there is a substantial lack of awareness of TB, especially in case of EPTB.

We speculate whether the diagnostic delay might have been avoided in our case with an elaborate anamnesis up front, accounting for ethnicity—noting the patient's origin from a high-endemic area—relevant exposures including infectious diseases among relatives, and concomitant symptoms of fever, night sweats, and weight loss, as this information should raise the suspicion of TB/EPTB. Early diagnosis and early onset of treatment are crucial for minimizing morbidity and mortality in spinal TB [4, 10], and in our case, acute surgery might have been avoided if the diagnosis was suspected up front.

In conclusion, this case demonstrates the potential consequences of diagnostic delay in spinal TB. It underlines the importance of a thorough patient history up front, accounting for ethnicity, exposure, and concomitant symptoms, and is a reminder that TB, including EPTB, should be considered as a differential diagnosis in patients from high-endemic areas.

## Data Availability

No underlying data were collected or produced in this case report, and data supporting this case report are from previously reported studies and reports, which have been cited.
